# The Determination of Flare-Up Incidence and Associated Risk Factors During Endodontic Treatment: An Observational Retrospective Study

**DOI:** 10.7759/cureus.31424

**Published:** 2022-11-12

**Authors:** Shilpa S Magar, Afrah Yousef Alfayyadh, Khluod Khalifah Alruwaili, Hessah Fahad F Almunahi, Ahmed Hamoud L Alsharari, Shaliputra P Magar

**Affiliations:** 1 Department of Conservative Dentistry and Endodontics, College of Dentistry, Jouf University, Sakaka, SAU; 2 Department of Oral Medicine, College of Dentistry, Jouf University, Sakaka, SAU; 3 Department of Oral Medicine and Radiology, College of Dentistry, Jouf University, Sakaka, SAU

**Keywords:** risk factors, pain, flare-up management, flare‑up, endodontic treatment

## Abstract

Background: Endodontic flare-up signifies pain and/or swelling seen within a few days after an endodontic appointment by patients requiring an emergency. These are undesirable and unwanted as they cause great discomfort to the subjects and stress to the operator due to swelling and pain.

Aims: This study aims to determine the flare-up incidence and associated risk factors during endodontic treatment.

Methods: The present retrospective study assessed 1000 patients of both genders for endodontic flare-ups. Flare-up cases were patients having swelling or pain within 1-3 days after a root canal appointment and needing an emergency visit for relieving the symptoms. After data recording, it was subjected to statistical analysis to assess related factors, flare-up cause, and incidence rate using Fisher's exact test and chi-square test.

Results: In the present study, the incidence rate of flare-ups is 9.4%. The majority of flare-ups were in the molar teeth followed by the anterior teeth and 6.7% (n=30) of the premolar teeth. For the number of canals, it was seen in 13.6% (n=24) of cases with multiple canals, 5.5% (n=12) of cases with two canals, and 9.6% (n=58) of cases with a single canal. In patients with no medical history, flare-ups were significantly less compared to patients with medical history (p<0.001). A significantly higher number of flare-ups were in the teeth having pulp necrosis with periapical lesions with 45.9% (n=34) (p<0.001).

Conclusion: Flare-ups are commonly seen in multiple canal teeth having pulp necrosis with periapical lesions with an associated medical history, with females being more prone to endodontic flare-up cases.

## Introduction

The chemo-mechanical method of disinfecting the root canal before three-dimensional hermetic obturation is termed endodontic treatment. Endodontic treatment is done to eliminate the peri-radicular and pupal pathologies and to increase tissue recovery in the peri-radicular region. Inter-appointment flare-ups are the most common and major problems faced by endodontists, despite newer instruments and techniques being introduced in endodontics [1]. Endodontic flare-up signifies pain and/or swelling seen within a few days after an endodontic appointment by patients requiring an emergency not scheduled to get relief of the associated symptoms [2].

These endodontic flare-ups are undesirable and unwanted as they cause great discomfort to the subjects and stress to the operator due to swelling and pain. The main etiology behind endodontic flare-ups is unknown. However, the most accepted etiology is multifactorial [3]. A positive correlation has been established in assessing the association of treatment protocol, the number of appointments, the operator's skill, preoperative symptoms and signs, periapical and pulpal conditions, tooth position, gender, and the age of the subject. For the management and prevention of endodontic flare-ups, the identification of the causative factors is vital. This helps in making appropriate treatment plans and reducing the chances of flare-up based on the individual tooth [4].

The incidence of endodontic flare-up ranges from 1.4% to 16% and can go up to 50% in a few subjects. The employment of preventive strategies for endodontic flare-ups seems to be the most appropriate measure to avoid their incidence [5]. It is vital to assess the etiology of flare-ups to manage them appropriately. However, flare-up has been associated with multifactorial etiology including different factors from the host: chemical, mechanical, and microbial etiologies. Other associated factors are related to the treatment including endodontic infections [6].

A correlation has been established between endodontic flare-up and the pulpal status of the tooth. Flare-ups are usually not seen with the teeth having inflamed or normal pulp if treatment is done under sterile and septic conditions with thorough debridement [7]. More flare-up incidents are reported in the teeth where previous endodontic treatment is done or was started and not completed. Concerning age, in subjects of age 50 years or above, more flare-up incidences are reported. Despite previous studies, the associated factors and incidence rates vary widely based on the protocol followed, treatment modalities, and population [8]. Hence, the present study was done to assess the endodontic flare-up incidence and to assess the associated risk factors.

## Materials and methods

The present retrospective study was done to determine the flare-up incidence and associated risk factors during endodontic treatment. The study was done at the Endodontic Section, College of Dentistry, Jouf University, Sakaka, Al-Jouf, Kingdom of Saudi Arabia; after, clearance was taken from the Institutional Ethical Committee with number IEC/07/08/1443h. The study data were obtained from the Department of Conservative Dentistry and Endodontics.

The inclusion criteria for the study were subjects in the age range of 18-60 years, subjects who underwent non-surgical endodontic treatment, and subjects whose complete clinical data were available as required for the study. The exclusion criteria for the study were subjects who were immunocompromised, pregnant and lactating females, and subjects with medical illness or systemic diseases. The study was based on the data collection extracted from the previous dental records, and no interaction was done with the subject, and the identity was also not taken into consideration.

Data were collected from the existing records of the subject's file from 2019 to 2021 for the subjects who got their root canal completed by the same endodontist with complete clinical data and records and considering the inclusion and exclusion criteria. After the final inclusion, the data collected from the subjects were gender, age, tooth type, tooth position, number of root canals, treatment type (previously initiated, initial, or retreatment), number of visits needed, pulpal and peri-radicular diagnosis of the tooth before the start of root canal treatment and status of pulp (vital/nonvital), preoperative radiograph for pathology assessment, postoperative radiograph for errors/obturation quality, root canal method (rotary/manual), irrigating solution used, whether the patient reported with/without a flare-up, flare-up symptom, and treatment given for flare-up.

Flare-up cases were subjects having swelling or pain within 1-3 days after a root canal appointment and needing an emergency visit to the dentist for relieving the symptoms. Subjects with no flare-up were those having no record of inter-appointment visits within 1-3 days of root canal completion. After data recording, it was subjected to statistical analysis to assess related factors, flare-up cause, and incidence rate using Fisher's exact test and chi-square test.

## Results

The study included a total of 1000 patients of both genders within the age range of 18-68 years who underwent endodontic treatment. In the present study, the incidence rate of flare-ups is 9.4%. The associated risk factors of the study subjects are listed in Table 1.

**Table 1:** Demographic and disease characteristics of the study subjects (p>0.05, not significant)

Characteristic	Total	Number of flare-ups (%)	P-value
Age (years)			
<20	74	2 (2.7)	0.058
20-29	362	46 (12.7)	
30-39	286	24 (8.4)	
40-49	218	18 (8.3)	
50-59	46	4 (8.7)	
≥60	14	0 (0.0)	
Gender			
Male	538	49 (9.1)	0.733
Female	462	45 (9.7)	

There were 538 males and 462 females in the present study where flare-up was noted in 9.1% (n=49) of males and 9.7% (n=45) of females. The difference was statistically non-significant with p=0.733. Most of the study subjects were in the age range of 20-29 years with 362 subjects followed by 286 subjects in 30-39 years, 2018 subjects in 40-49 years, 74 in <20 years, 46 subjects in 50-59 years, and 14 subjects in ≥60 years. Endodontic flare-up was most common in 20-29 years of age with 12.7% (n=46) of subjects followed by 8.7% (n=4) in 50-59 years, 8.4% (n=24) in 30-39 years, 8.3% (n=218) in 40-49 years, 2.7% (n=2) in <20 years, and in no subject of age ≥60 years (Table 1).

On assessing the factors associated with endodontic flare-ups, it was seen that for the tooth, the majority of flare-ups were in the molar teeth with 14.3% (n=28) of cases followed by 10.1% (n=356) of flare-ups in the anterior teeth with p=0.008 and 6.7% (n=30) of the premolar teeth. For the number of canals, it was seen in 13.6% (n=24) of cases with multiple canals, 5.5% (n=12) of cases with two canals, and 9.6% (n=58) of cases with a single canal. For the number of canals, it was significant with p=0.02. Concerning treatment, the majority of flare-ups were in initial endodontic treatment cases with 9.7% (n=86) of subjects (p=0.143), 18.2% (n=4) and 5.1% (n=4) of subjects in re-endodontic treatment and previously initiated endodontic treatment cases, and in no subject with intentional endodontic treatment cases. On assessing the medical history, in subjects with no medical history, flare-ups were significantly less in 8.1% (n=78) of subjects compared to subjects having medical history in 38.1% (n=16) (p<0.001). Concerning diagnosis, the significantly higher number of flare-ups was in subjects having pulp necrosis with periapical lesion with 45.9% (n=34) followed by pulp necrosis with normal periapical tissue in 16.7% (n=20) of subjects, 5.6% (n=18) and 5.2% (n=18) of subjects each having asymptomatic irreversible pulpitis and symptomatic irreversible pulpitis, and 2.9% (n=4) of subjects with chronic irreversible pulpitis (p<0.001). In radiographic findings, 50% (n=2) of subjects with thickened lamina dura showed flare-up, 6.8% (n=44) of subjects with normal periapical tissues, 20.5% (n=30) of subjects with abscess, and 8.7% (n=18) of subjects with discontinuation of the lamina dura. This was statistically significant with p<0.001. For treatment, the flare-ups were statistically non-significant with p=0.143. Pulp vitality was seen in 344 subjects, and nonvitality was observed in a total of 656 teeth with different diagnoses as mentioned in Table 2.

**Table 2:** Factors associated with flare-ups in the study subjects

Factors		Total	Number of flare-ups (%)	P-value
Tooth	Anterior	356	36 (10.1)	0.008
	Premolar	448	30 (6.7)	
	Molar	196	28 (14.3)	
Root canal	Single	604	58 (9.6)	0.021
	Two canals	220	12 (5.5)	
	Multiple canals	176	24 (13.6)	
Treatment	Initial endodontic treatment	884	86 (9.7)	0.143
	Previously initiated endodontic treatment	78	4 (5.1)	
	Re-endodontic treatment	22	4 (18.2)	
	Intentional endodontic treatment	16	0 (0.0)	
Medical history	No medical history	958	78 (8.1)	<0.001
	Medical problem	42	16 (38.1)	
Diagnosis	Chronic irreversible pulpitis (nonvital)	138	4 (2.9)	<0.001
	Symptomatic irreversible pulpitis (vital)	344	18 (5.2)	
	Asymptomatic irreversible pulpitis (nonvital)	324	18 (5.6)	
	Pulp necrosis with normal periapical tissue (nonvital)	120	20 (16.7)	
	Pulp necrosis with periapical lesion (nonvital)	74	34 (45.9)	
Radiographic finding	Normal periapical tissue	644	44 (6.8)	<0.001
	Abscess	146	30 (20.5)	
	The discontinuation of the lamina dura	206	18 (8.7)	
	Thickened lamina dura	4	2 (50.0)	

Among the 1000 subjects assessed, flare-up was reported in 94 study subjects. Concerning visits, 70.2% (n=66) of flare-ups were seen in three visits followed by 17% (n=16) in three visits and 12.8% (n=12) in one visit. For signs and symptoms, moderate to severe pain was reported in 53.2% (n=50) of subjects, pain and swelling in 36.2% (n=34) of subjects, and only swelling in 10.6% (n=10) of study subjects. Emergency care was needed in 30.9% (n=29) of subjects and was not needed in 69.1% (n=65) of subjects with flare-ups. On assessing the etiology of the flare-up, the most common possible cause was found to be nonvital tooth (bacterial) in 54.3% (n=51) of subjects followed by 21.3% (n=20) of subjects, incomplete pulp extirpation in 18.1% (n=17) of subjects, and chemical cause in 6.4% (n=6) of study subjects as shown in Table 3.

**Table 3:** Parameters of the endodontic flare-ups in the study subjects

Parameters	Subgroup	Number (n=94)	Percentage (%)
Visits	One	12	12.8
	Two	66	70.2
	Three	16	17.0
Signs and symptoms	Moderate to severe pain	50	53.2
	Pain and swelling	34	36.2
	Only swelling	10	10.6
Emergency	Needed	29	30.9
	Not needed	65	69.1
Possible causes	Incomplete pulp extirpation	17	18.1
	Chemical	6	6.4
	Mechanical	20	21.3
	Nonvital tooth (bacterial)	51	54.3

## Discussion

Flare-ups are commonly reported after endodontic treatment despite advances in techniques and instruments of root canal treatment as the skill of the treating personnel is judged by the ability to relieve pain and associated symptoms such as swelling. Multifactorial etiology has been associated with flare-ups. These are undesirable, and prediction can be done if risk factors are identified in a subject. The present study was retrospective, which prevents bias by the investigator and operator when contrasted to the prospective studies. However, certain limitations have been linked to retrospective studies including random case selection and relying on secondary data, whereas minimum expense and larger sample size can be used in the prospective studies, which are not seen in the retrospective studies as suggested by Iqbal et al. [9] in 2009.

The present study assessed flare-up as the outcome of the endodontic treatment with the consideration of various factors including tooth type, number of canals, treatment modality, medical history, diagnosis, and radiographic findings where significant findings were seen for tooth type, canal number, medical history, diagnosis, and radiographic findings with respective p-values of 0.008, 0.02, <0.001, <0.001, and <0.001, whereas for treatment type, the results were non-significant. Concerning treatment, the majority of flare-ups were in initial endodontic treatment cases with 9.7% (n=86) of subjects, 18.2% (n=4) and 5.1% (n=4) of subjects in re-endodontic treatment and previously initiated endodontic treatment cases, and in no subject with intentional endodontic treatment cases. On assessing the medical history, in subjects with no medical history, flare-ups were significantly less with 8.1% (n=78) of subjects compared to subjects having medical history in 38.1% (n=16) (p<0.001). Concerning diagnosis, the significantly higher number of flare-ups was in subjects having pulp necrosis with periapical lesion with 45.9% (n=34) followed by pulp necrosis with normal periapical tissue in 16.7% (n=20) of subjects, 5.6% (n=18) and 5.2% (n=18) of subjects each having asymptomatic irreversible pulpitis and symptomatic irreversible pulpitis, and 2.9% (n=4) of subjects with chronic irreversible pulpitis (p<0.001). In radiographic findings, 50% (n=2) of subjects with thickened lamina dura showed flare-up, 6.8% (n=44) of subjects with normal periapical tissues, 20.5% (n=30) of subjects with abscess, and 8.7% (n=18) of subjects with discontinuation of the lamina dura. This was statistically significant with p<0.001. For treatment, the flare-ups were statistically non-significant with p=0.143. For the number of canals, it was significant with p=0.02. These results were consistent with the studies by Onay et al. [10] in 2015 and Azim et al. [11] in 2017 where the authors reported the significant association of canal number, radiographic diagnosis, and treatment modality in their studies as in the present study.

Concerning the demographic characteristics, maximum flare-ups were seen in 20-29 years of age followed by 50-59 years and 30-39 years, which were significant with p=0.05. The majority of the study subjects were in the age range of 20-29 years with 362 subjects followed by 286 subjects in 30-39 years, 2018 subjects in 40-49 years, 74 in <20 years, 46 subjects in 50-59 years, and 14 subjects in ≥60 years. Endodontic flare-up was most common in 20-29 years of age with 12.7% (n=46) of subjects followed by 8.7% (n=4) in 50-59 years, 8.4% (n=24) in 30-39 years, 8.3% (n=218) in 40-49 years, 2.7% (n=2) in <20 years, and in no subject of age ≥60 years. This was in contrast with Ozdemir et al. [12] in 2010 where the authors reported the highest incidence of the flare-up following endodontic treatment in subjects of higher age at 50 years and more. Also, in the present study, a higher incidence of flare-up was reported in females compared to the males, which was statistically non-significant with p=0.733. These findings were similar to the studies by Vera et al. [13] in 2012 and Siqueira and Barnett [14] in 2004 where the authors reported a higher incidence of flare-up in females compared to males. This can be attributed to the alteration in the hormonal levels of females, oral contraceptives, or hormone replacement therapy, which can change noradrenaline and serotonin levels leading to a decreased pain threshold.

For the assessment of the parameters of the endodontic flare-up, second visit was maximum. The most common associated sign and symptoms were moderate to severe pain; emergency management was not needed in 69.1% (n=65) of subjects, and the most common suspected etiologic factor associated was bacteria seen in the nonvital tooth in 54.3% (n=51) of study subjects. These findings were in agreement with the studies of Schwendicke and Göstemeyer [15] in 2017 and Mathew [16] in 2015 where the authors reported similar flare-up parameters as in the present study. The study limitations are very limited sample size and the etiology of flareup not being recorded when the inclusion was mentioned. The study did not check for the microbial analysis of the lesions, and hence, further studies can be taken in future.

## Conclusions

Considering its limitations, in the present study, the incidence rate of flare-ups is 9.4%; the present study concludes that flare-ups are commonly seen in multiple canal teeth having pulp necrosis with periapical lesions with an associated medical history, with females being more prone to the endodontic flare-up cases. The most associated etiology was bacteria in nonvital teeth, and pain was the most common associated symptom. However, more longitudinal and prospective studies are needed to identify the associated risk factors for endodontic flare-ups including medicament used during treatment, irrigants, microorganism's role, and the medical condition of the subjects.
